# Mechanical Characterization of Graphene-Enhanced Fiber Rope Composites for Strengthening-Oriented Applications

**DOI:** 10.3390/polym17172304

**Published:** 2025-08-26

**Authors:** Ahmet E. Haberdar, Volkan Acar, Ferit Cakir

**Affiliations:** 1 Department of Civil Engineering, Gebze Technical University, 41400 Kocaeli, Türkiye; cakirf@gtu.edu.tr; 2 Department of Mechanical Engineering, Ataturk University, 25240 Erzurum, Türkiye; volkanacar@atauni.edu.tr

**Keywords:** graphene nanoplatelets, fiber rope composites, mechanical testing, structural strengthening, material efficiency

## Abstract

Achieving high mechanical performance in fiber-reinforced composites is essential for developing reliable and sustainable strengthening systems that aim to enhance service life and reduce the waste of resources. In particular, fiber rope composites, with their inherent flexibility and excellent structural properties, offer significant potential as reinforcement elements in strengthening applications. The mechanical properties of these composites could be further enhanced using a remarkably basic and fundamental method. In this study, this fundamental and effective method, nanoparticle modification, is presented at its most basic level. This research presents an experimental investigation into the mechanical behavior of 8 mm diameter carbon, basalt, and glass fiber rope composites, produced in both unmodified and graphene nanoplatelet (GNP)-modified forms. GNPs were reinforced into an epoxy matrix at weight fractions of 0.5%, 1%, and 2% to enhance the mechanical properties of the fiber rope composites. Fiber rope composites were fabricated using controlled mixing, molding, and curing techniques. Subsequently, a series of mechanical tests, including flexural, compressive, and buckling tests, were conducted to evaluate the impact of nanoparticle reinforcement on structural performance. The findings reveal that GNP modification leads to notable improvements in mechanical properties, suggesting that such enhanced composites may contribute to more resilient and long-lasting strengthening solutions. These results underscore the relevance of nanoparticle-enhanced composites in the context of material efficiency and end-of-life considerations in structural systems, particularly through extended usability and improved performance.

## 1. Introduction

Fiber-reinforced polymer composite materials are widely used in various industries, particularly aerospace and marine industries, due to their superior properties [1]. In addition to these fields, the use of these materials in the construction industry has advanced significantly in recent years, with notable progress in both laboratory research and field applications. Composites used in the field of construction are generally products containing a long or short fiber-reinforced polymer matrix. These products have proven themselves in the strengthening and restoration of structures. However, in some application areas in the field, reinforcements using long fibers/fabric may have various limitations due to reasons such as the geometry of the structure. Considering these situations, the need for different types of fiber reinforcement elements is increasing. Fiber ropes can be suitable components for such reinforcement scenarios. Moreover, their higher strength-to-weight ratio compared to conventional metallic wire ropes makes them interesting for reinforcement and construction [2].

Currently, the use of fiber ropes in construction is limited. While it is observed that they are primarily used in the strengthening of historical structures in practice, research studies on the subject are also limited. In this context, studies on strengthening masonry structures with basalt fiber rope can be given as an example [3,4]. Fiber ropes have also been used to strengthen reinforced concrete (RC), and laboratory tests have been conducted. Concrete columns and frames reinforced with basalt and polypropylene fiber rope [5], aramid fiber rope [6], or Kevlar–carbon hybrid rope [7] have been studied. These studies yielded positive results, demonstrating the efficiency of fiber ropes in strengthening reinforced concrete. On the other hand, fiber ropes produced from natural fibers have also been involved in strengthening studies. For this purpose, researchers have utilized coconut fiber rope [8,9], hemp fiber rope [10,11], cotton fiber rope [11], and jute fiber rope [12] in concrete and masonry structures.

The characterization of fiber ropes has revealed the dynamics in the mechanical behavior of these products. In a study conducted in this context, the tensile characterization of basalt fiber rope and rod was performed, and the tensile test protocol was examined [13]. To overcome the difficulties that may arise in the tensile testing of fiber ropes, the specimen preparation technique employed in this study can be considered for other synthetic fibers. In another study, the effects of the aging processes of basalt fiber rope exposed to water, acid, and alkaline environments on mechanical properties were investigated. Among these environments, aging in an alkaline environment was found to adversely affect the mechanical behavior of basalt fiber rope, and a suitable coating was recommended for the basalt fiber rope surfaces in such environments [14]. Coating application was carried out for carbon fiber rope, and it was reported that a copper-based coating improved the mechanical properties of the rope. In addition, the same study found that the use of rope was more advantageous in terms of strength than Al matrix composites reinforced using carbon rope and bundle fiber [15]. In addition to traditional synthetic fibers, the cyclic loading and bedding-in performance of high-modulus polyethylene fiber (HMPE) ropes have also been the subject of research [16,17].

Another primary process in this study is matrix modification by nanoparticle dispersion. Previous studies have reported that modifications, especially those involving carbon allotropes, have a positive effect on both the epoxy resin and the reinforcing fiber. It is known that nanoparticle integration increases the toughness and strength of the resin. Additionally, this reinforcement process enhances the fiber–matrix interface, and improved interface interactions lead to enhanced final mechanical properties of fiber-reinforced polymer composites [18]. In a study conducted on carbon fiber prepregs, the reinforcement of epoxy resin with graphene oxide enhanced the adhesion between the fiber and matrix and improved the mechanical behavior of the prepreg composites [19]. It is well known that the mechanical properties of epoxy resin dispersed with graphene nanoparticles (GNPs) are significantly improved [20,21]. Prepregs modified with graphene nanoparticles have also shown high performance in reinforcing masonry and concrete structures [22,23,24]. The improvements achieved in the strength and other mechanical properties of the composite with graphene reinforcement, compared to the composite without nanoparticle reinforcement, have a positive effect on the survival potential of the structure by increasingly helping to carry the load applied to the strengthened structure.

In strengthening applications, composite materials are required to be easily and practically applied to the structure. The use of solid rod composites in strengthening applications is limited due to the geometric constraints of the rod and the structure. Fiber rope composites, on the other hand, attract attention as an essential composite material type for these applications because of their flexibility and advanced wrapping capability. In this context, fiber rope composites were utilized in a strengthening study conducted by our research group, and it was understood that these composites are an efficient strengthening element for structures with irregular geometries [25].

In this study, an effective method is proposed to enhance the efficiency of fiber rope composites further using a fundamental and practical approach: nanoparticle modification. It is considered that wrapping flexible and dry fiber ropes immersed in nanoparticle-modified resin around the structure is a basic process that is quite easily applicable. Therefore, the results of this research are expected to bring a new perspective to the literature and field applications in terms of examining the mechanical efficiency of nanoparticle-reinforced fiber rope composites. In this study, GNPs were incorporated into the epoxy matrix at weight fractions of 0.5%, 1%, and 2% to enhance the mechanical performance of fiber-rope-based composite materials. The effects of GNP reinforcement on fiber rope–epoxy composites were investigated through density, flexural, compressive, and buckling tests, providing insights into their potential use in structural strengthening applications. The findings highlight the potential of nanoparticle-modified composite ropes for structural reinforcement applications, particularly in the context of lightweight, durable, high-strength materials for seismic retrofitting and civil engineering projects.

## 2. Materials and Methods

### 2.1. Materials

#### 2.1.1. Fiber Ropes

**Figure 1:**
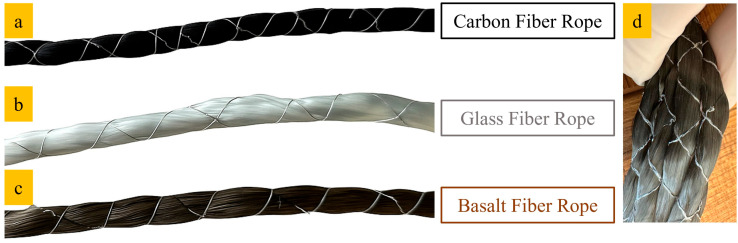
Fiber ropes used in the study: (**a**) carbon fiber, (**b**) glass fiber, (**c**) basalt fiber; (**d**) the flexibility of the dry ropes.

The composite ropes utilized in this research were fabricated from three types of fiber rope materials with a diameter of 8 mm: carbon, glass, and basalt (Figure 1). All ropes were supplied from Tekno Construction Chemicals (İstanbul, Türkiye).

#### 2.1.2. Epoxy System

The epoxy resin system was obtained from Teknobond 330, a product of Tekno Construction Chemicals (İstanbul, Türkiye). This system consists of two components, as specified in the technical data sheet provided by the manufacturer: Component A (epoxy resin) comprising 80% of the mixture and Component B (hardener) comprising 20% [26].

#### 2.1.3. Graphene Nanoplatelets (GNPs)

GNPs were supplied from Nanografi Co. (Ankara, Türkiye). The physical properties of the GNPs were provided by the supplier as follows: a thickness of approximately 5 nm, an average diameter of 18 µm, a surface area of 170 m^2^/g, and a purity of 99%.

### 2.2. Manufacturing of the Composite Ropes

#### 2.2.1. Manufacturing of Unmodified Fiber Rope Composites

**Figure 2:**
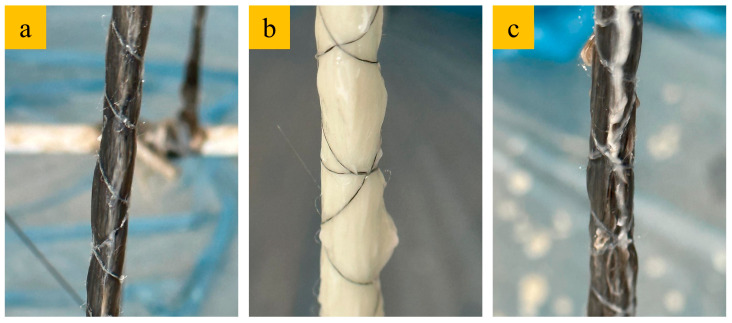
Wetting of the unmodified composite ropes; (**a**) CF, (**b**) GF and (**c**) BF.

The production of unmodified composite ropes followed a standard FRP manufacturing process. The epoxy system, composed of Teknobond 330, was prepared by mixing Component A (80%) and Component B (20%) using a mechanical mixer (R. Bosch GmbH, Germany). The mixture was stirred initially and then subjected to 400 rpm mechanical mixing for 6 min to ensure homogeneity. Following the preparation of the epoxy mixture, fiber ropes were immersed in the resin for 6 min, allowing thorough impregnation of the fibers. After impregnation, the resin-saturated fiber ropes were wrapped around a steel frame to form the desired composite shape (Figure 2). The wrapped composite ropes were then left to cure for one week under controlled conditions (at room temperature with stable relative humidity). After the curing period, the ropes were cut to standardized dimensions and prepared for mechanical testing, including tensile, flexural, compressive, and buckling tests.

#### 2.2.2. Manufacturing of GNP-Modified Fiber Rope Composites

**Figure 3:**
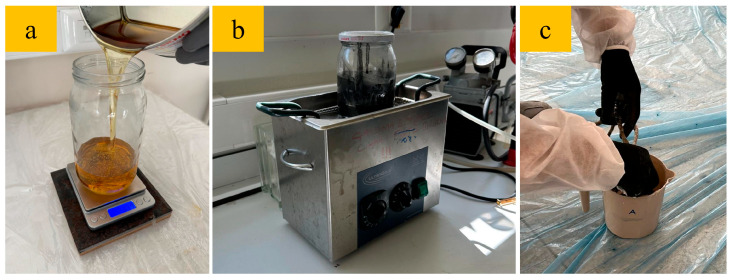
Preparation of GNP–epoxy resin system: (**a**) weighing and (**b**) ultrasonic mixing of GNP/hardener mixture and (**c**) the final mixture of GNP–hardener–epoxy resin prepared for the wetting of fiber ropes.

The production process of nanoparticle-modified composite ropes followed a similar sequence to that of the unmodified specimens, with additional steps for incorporating and dispersing GNPs. The nanoparticles were added to Component B (hardener) at ratios of 0.5%, 1%, and 2% by weight of the final epoxy resin system to ensure precise dispersion of the material. A uniform dispersion is necessary to achieve an efficient matrix modification that directly impacts the structural performance of nanocomposites [27]. To achieve a homogeneous dispersion, the initial mixture was first manually stirred and then subjected to mechanical mixing at 400 rpm for 6 min, ensuring homogeneity before ultrasonic treatment. Following this, the mixture was subjected to ultrasonic processing in an ultrasonic bath (Hydra Ultrasonic, İstanbul, Türkiye) for 15 min at 500–2000 W, which further increased the distribution of nanoparticles in the polymer matrix (Figure 3). Once the nanoparticle-modified resin mixture was prepared, it was combined with the Component A, thoroughly mixed mechanically, and immediately used for fiber impregnation. The composite ropes were immersed in the nanoparticle-modified resin for 6 min, ensuring complete saturation. They were then wrapped onto a steel skeleton, similar to the unmodified specimens, and left to cure for one week at room temperature with stable relative humidity. After curing, the nanoparticle-modified composite ropes were cut and prepared for standardized mechanical testing, allowing direct comparison with the unmodified samples.

### 2.3. Density Measurements

**Figure 4:**
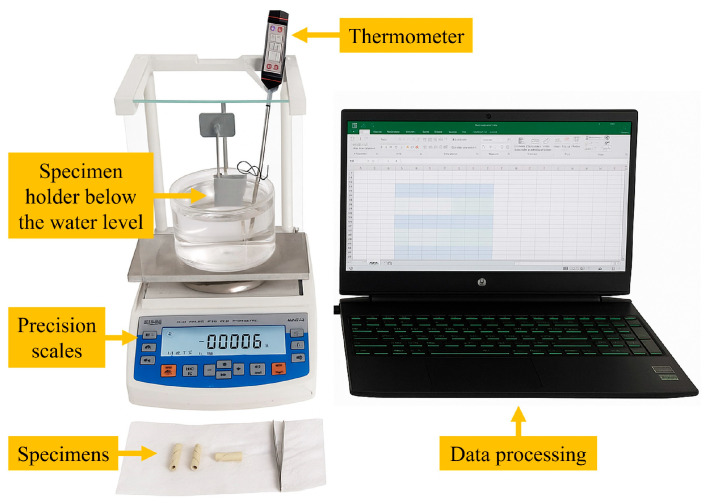
Schematic of the density measurement setup.

Density measurements were performed using the experimental setup (AS 220.R2, Radwag, Poland) established in accordance with the ASTM D792 standard [28]. The density of the fiber rope composites was calculated by averaging three repeated measurements taken from three specimens of each type of rope. Figure 4 shows the experimental setup used for the relevant standard.

### 2.4. Mechanical Tests

**Figure 5:**
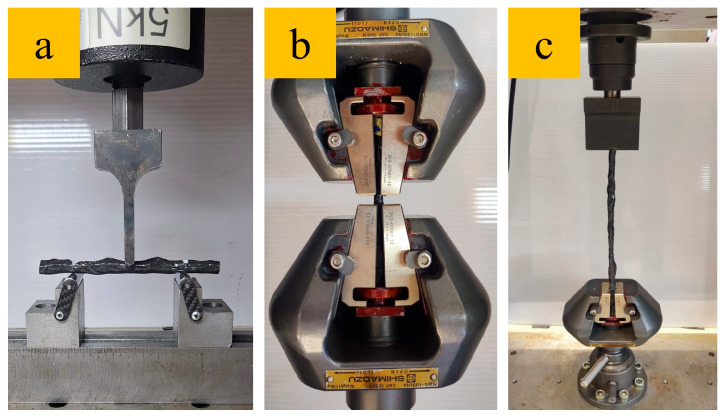
Mechanical tests: (**a**) three-point bending, (**b**) compression, and (**c**) buckling.

To evaluate the mechanical performance of the composite ropes, a series of standardized mechanical tests was conducted. These tests, shown in Figure 5, assessed the flexural, compressive, and buckling properties of both nanoparticle-modified and unmodified specimens. The aim was to determine the impact of GNP reinforcement on the structural behavior of the composite materials. A universal mechanical test device (AG-IS, Shimadzu Corp., Kyoto, Japan) with load cells of 5 kN and 100 kN was used for the mechanical tests.

#### 2.4.1. Flexural Test

The flexural behavior of the composite ropes was examined using three-point bending tests. The tests were conducted on specimens with a support span-to-depth ratio of 8:1 at a test speed of 1 mm/min. The flexural strength and displacement behavior of the specimens were analyzed to compare the bending resistance of nanoparticle-modified and unmodified composite materials. At least six specimens were used for the flexural tests.

#### 2.4.2. Compression Test

To assess the compressive strength of the composite ropes, tests were conducted using the relevant device on at least six specimens. The loading was applied at a controlled displacement rate of 1 mm/min until failure. The compressive strength and failure mechanisms were recorded to evaluate the influence of nanoparticle modification on the load-bearing capacity of the ropes under compression.

#### 2.4.3. Buckling Test

Buckling tests were conducted to assess the stability and resistance of composite ropes under axial loads on at least six specimens. The specimens were subjected to uniaxial compression with one end free and the other end fixed, simulating real-world conditions. The critical buckling load and the corresponding displacement were measured to assess the enhancement in the load-bearing capacity and stability resulting from GNP reinforcement.

### 2.5. Fracture Analysis and Visual Inspection

Following the mechanical tests, to gain a deeper understanding of the fracture process, failure mode, bonding mechanism, and crack morphology, high-resolution digital microscope (DM) examinations were conducted. A digital microscope (Bushman 1600X, China) was used for visual inspections. For analysis, failed specimens from mechanical testing were sectioned at the fractured surfaces. The cross-sections were carefully examined under the digital microscope, allowing for the detailed observation of fiber–matrix bonding, potential debonding zones, and crack propagation patterns. These visual inspections provided critical insights into the failure mechanisms of both nanoparticle-modified and unmodified composite ropes, highlighting differences in fracture behavior due to GNP reinforcement.

## 3. Results

### 3.1. Density Results

**Figure 6:**
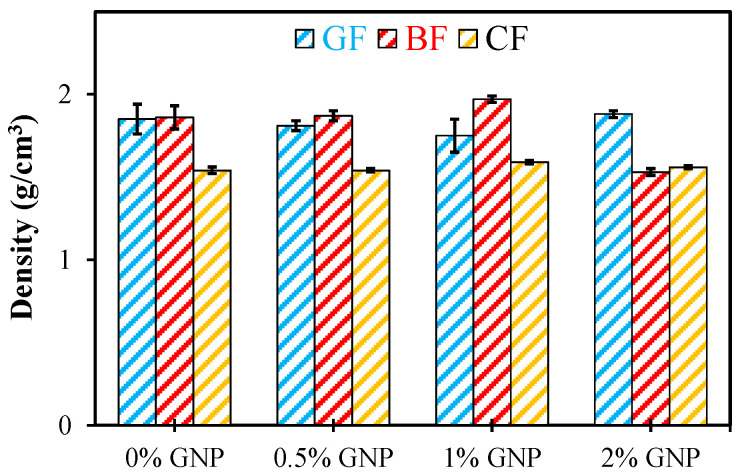
Density results of glass fiber (GF), basalt fiber (BF), and carbon fiber (CF) rope composites.

The densities of fiber rope-based composites are given in Table 1. Accordingly, the low density of the carbon fiber rope among the control specimens is noteworthy. Similar values were observed in the densities of glass and basalt fibers. While there was no significant change in the density values of the glass and carbon fiber rope based composites with GNP reinforcement, a significant decrease occurred in the basalt fiber rope composites with the 2% GNP reinforcement. The results are also shown in Figure 6.

### 3.2. Mechanical Test Results

**Figure 7:**
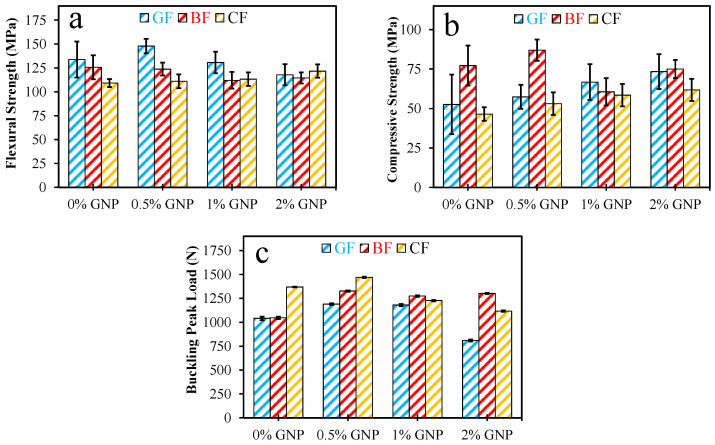
Mechanical properties of composite ropes: (**a**) average flexural strength, (**b**) average compressive strength, and (**c**) average buckling peak load.

The experimental study was designed to evaluate the mechanical performance of composite ropes manufactured with carbon, basalt, and glass fibers incorporating GNPs at 0.5%, 1%, and 2% weight fractions. The study aimed to evaluate how these nano-reinforcements affect the behavior of the composites under various load conditions by subjecting the composites to tensile, flexural, compressive, and buckling tests. In general, the experiments revealed that optimum and moderate levels of GNP addition increased the load-carrying capacity and durability of the composites, while excessive nanoparticle content resulted in adverse effects due to agglomeration and increased resin viscosity. Tensile test results showed that a low GNP concentration (approximately 0.5%) resulted in a significant improvement in flexural strength. At this level, the nanoparticles were uniformly dispersed in the epoxy matrix, promoting robust interfacial bonding and facilitating efficient load transfer between the fibers and the matrix. When the GNP content increased to 1% or 2%, the nanoparticles tended to aggregate, leading to localized stress concentration points that hindered effective load transfer. As a result, the tensile strength decreased compared to the optimally reinforced composites. The comparison bar chart format is shown in Figure 7.

The flexural tests demonstrated that the flexural performance of the composites also benefited from an optimal reinforcement of the GNP. At approximately 0.5% GNP, well-dispersed nanoparticles formed a reinforcement network within the matrix, thus increasing the flexural stiffness and effectively delaying crack propagation under flexural loads. However, when the nanoparticle content exceeded this modest level, aggregation occurred, disrupting the continuity of the matrix. This phenomenon reduced the ability of the composites to evenly distribute flexural stresses evenly, ultimately resulting in a decrease in flexural strength.

The compressive strength tests revealed a similar trend, where low doses of GNPs increased the resistance of the composites to compressive forces. Evenly distributed nanoparticles strengthened the matrix and delayed the onset of microcracking under compression. When the GNP content remained within the optimum range, the compressive strength improved significantly. In contrast, at higher GNP levels, the formation of local clusters acted as defects within the matrix, thereby slightly weakening the material’s ability to distribute compressive stresses effectively. The buckling tests provided further evidence of the critical role played by the GNP content in providing structural stability. At an optimum concentration of approximately 0.5%, improved interfacial bonding and increased stiffness contributed to a higher buckling load, which showed greater resistance to axial compression. However, with excessive nanoparticle incorporation, increased resin viscosity, and the appearance of nanoparticle clusters, the uniformity of stiffness was compromised. This inconsistency led to a decrease in buckling capacity as the material became more susceptible to early local deformations. The experimental results are presented in Table 2, Table 3 and Table 4. In summary, the experimental findings showed that the controlled, moderate addition of GNPs was beneficial for improving the mechanical properties of composite ropes. The optimum nanoparticle concentration provided uniform distribution and effective load transfer, which significantly improved tensile, flexural, compressive, and buckling performances. In contrast, excessive GNP addition led to agglomeration and increased resin viscosity, negatively affecting the structural performance of the composites. These insights provided a solid foundation for tailoring nanoparticle-reinforced composites to meet specific engineering requirements, particularly in applications involving structural reinforcement and seismic strengthening.

**Figure 8:**
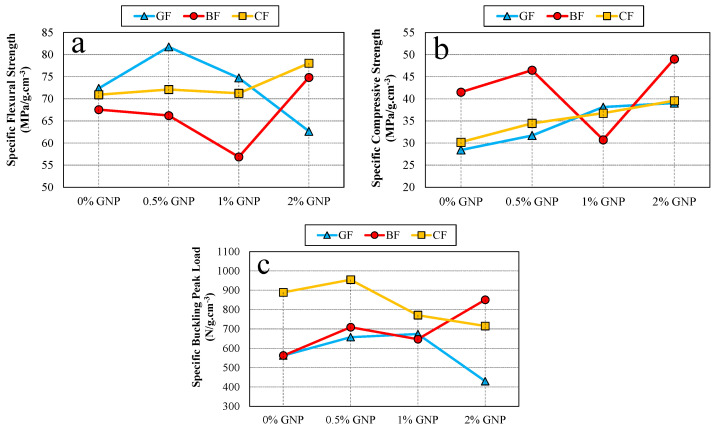
Specific strength and load variations depending on graphene content: (**a**) specific flexural strength, (**b**) specific compressive strength, and (**c**) specific buckling peak load.

**Figure 9:**
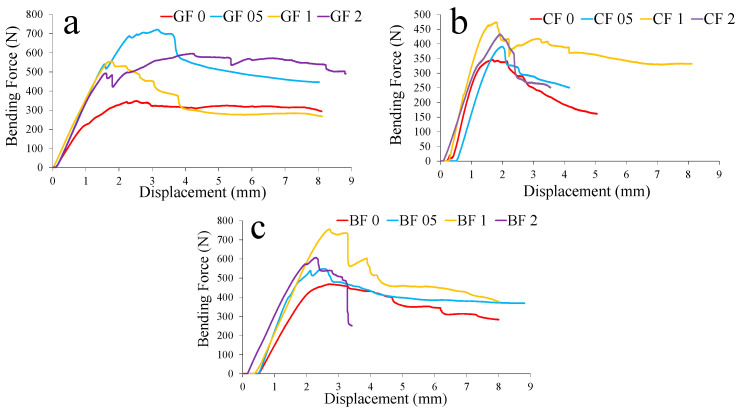
Representative bending force–displacement curves: (**a**) glass fiber (GF), (**b**) carbon fiber (CF), and (**c**) basalt fiber (BF) rope composites.

**Figure 10:**
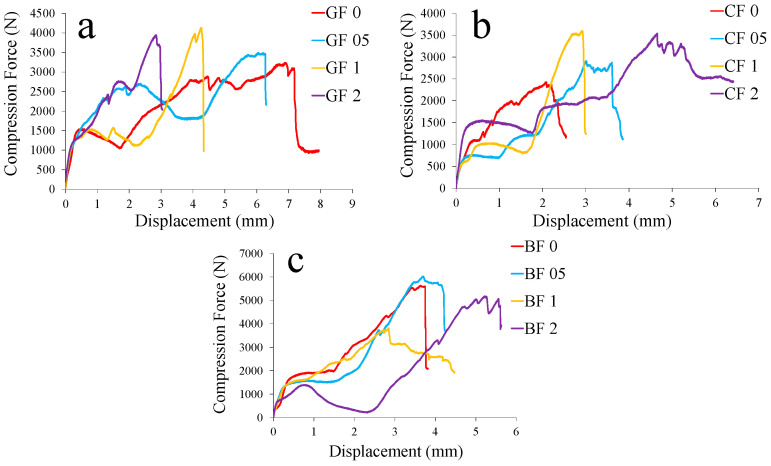
Representative compression force–displacement curves: (**a**) glass fiber (GF), (**b**) carbon fiber (CF), and (**c**) basalt fiber (BF) rope composites.

**Figure 11:**
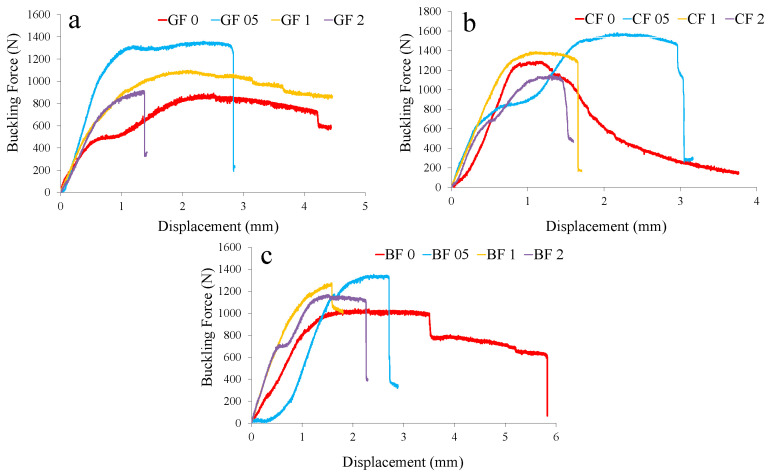
Representative buckling force–displacement curves: (**a**) glass fiber (GF), (**b**) carbon fiber (CF), and (**c**) basalt fiber (BF) rope composites.

Figure 8 shows the specific strength and buckling load changes of rope fiber composites depending on graphene content. In Figure 8a, the positive performance of glass fiber rope at a 0.5% reinforcement ratio in terms of specific flexural strengths is remarkable, while it reaches the lowest specific strength value at a 2% reinforcement ratio. High specific flexural strength values were observed for basalt and carbon fiber ropes at the 2% reinforcement ratio. For specific compressive strength, increases occurred for all fibers at 0.5% and 2% reinforcements (Figure 8b). The improved values at a 0.5% reinforcement ratio are also noteworthy for the specific buckling strengths. For this test, the 2% reinforcement ratio increased only for basalt fiber rope composites (Figure 8c). Figure 9, Figure 10 and Figure 11 show representative force–displacement curves from the data obtained in all mechanical tests.

**Figure 12:**
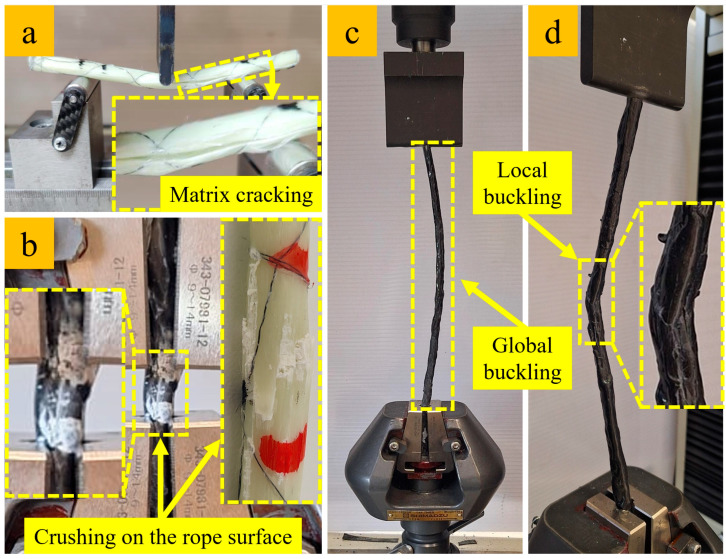
Failure mechanism of fiber rope composites: (**a**) flexibility-induced deflection and matrix cracking in the flexural test, (**b**) crushing on the rope surface in the compression test, (**c**) global buckling, and (**d**) local buckling.

Figure 12 shows the fracture mechanisms of the fiber rope composites. As shown in Figure 12a, the unwetted regions in the cores of fiber ropes tend to bend under lower loads during the flexural test. Additionally, matrix cracking on the surface is frequently observed in the failure mechanism of flexural specimens. Figure 12b shows the crushing observed on the surfaces of the fiber ropes during the compression test. The fractures that resulted from the deformation of the shell structure on the surface due to the increasing compression force occurred in the form of crushing. Figure 12c shows the global buckling behavior affecting the entire length of the test specimen recorded during the buckling test, while Figure 12d shows the local buckling failure. These two types of buckling behaviors are directly related to the wall thickness of the wetted shell surface within the cross-section of the fiber rope composite. An increase in the wall thickness of the shell surface leads to global buckling behavior, while a decrease in thickness causes the dry fibers to dominate, resulting in local buckling failure.

### 3.3. Visual Fracture Observations

In this experimental study, three different types of fiber ropes (glass, carbon, and basalt) were reinforced with GNP-modified epoxy resin at concentrations of 0.5%, 1%, and 2%. Visual and microscopic examinations revealed a consistent trend in all types of ropes: the outer layers of the ropes were effectively impregnated by the nanoparticle-reinforced resin, while the inner regions showed significant resin starvation and poor wetting. Among the materials tested, the glass fiber ropes showed the highest resin absorption capacity and relatively deeper penetration of epoxy into the inner layers. In contrast, the basalt fiber ropes exhibited the poorest impregnation behavior, with large sections of the core remaining dry and unbound. The carbon fiber ropes demonstrated intermediate performance characterized by better external encapsulation but still exhibited insufficient saturation in the core. These differences are probably due to the specific surface properties, weave densities, and capillary actions of each fiber type.

In all cases, the presence of GNPs in the resin enhanced the surface interaction between the matrix and the outermost fiber filaments, resulting in a robust outer shell. However, as the nanoparticle content increased, the resin viscosity also increased, hindering its ability to infiltrate the tightly packed inner filaments. Even at the lowest concentration of 0.5%, some core regions remained poorly impregnated, indicating that the rope architecture and process parameters play a more dominant role in resin penetration than nanoparticle content alone. These results highlight a critical challenge in rope-based composite systems: achieving homogeneous resin distribution across the entire cross-section remains challenging, especially when nanoparticle-modified matrices are used. This heterogeneity can compromise the mechanical integrity of the composite under loading, as the dry core fibers make a limited contribution to load transfer and are prone to early failure mechanisms, such as delamination, fiber pull-out, or internal cracking.

Samples containing 0.5% GNP reinforcement show significantly improved resin impregnation behavior compared to 1% and 2% GNP samples. These images show deeper penetration of the epoxy matrix into the fiber bundles and a more uniform distribution of the resin across the cross-section. The boundaries between the resin and fibers are less pronounced, and the number of dry, unbonded filaments is significantly reduced, indicating improved fiber–matrix adhesion and improved wetting performance. This is likely due to the lower nanoparticle content, which minimizes the increase in resin viscosity and thus provides better flow properties and capillary penetration into fiber tows. As a result, mechanical locking is more effective, which should contribute to superior load transfer and a reduced likelihood of premature delamination or fiber pull-out during stress application. However, despite this overall improvement, some internal regions still show signs of incomplete saturation, especially in the core. These areas constitute potential planes of weakness under loading, indicating that even at low nanoparticle loading, complete impregnation remains a challenge. However, from a comparative perspective, a GNP content of 0.5% represents the optimal balance between improved matrix properties and sufficient resin flow for composite integrity.

The mechanical test results of carbon fiber rope specimens reinforced with 1% GNPs reveal several critical issues related to resin impregnation and internal structural integrity. While the outer layers of the ropes appear to be sufficiently saturated with graphene-reinforced epoxy resin—forming a shiny and adhesive shell—the inner core exhibits serious deficiencies. A clear distinction is observed between well-impregnated surface fibers and dry, loosely bound filaments inside the core. The resin could not penetrate effectively through the entire cross-section, possibly due to high viscosity, poor wetting behavior, or limited capillary action within the tightly packed carbon fibers. This resulted in widespread resin starvation zones where dry fibers remained unbonded, leading to poor fiber–matrix interaction. As a result, mechanical failure was governed by delamination, fiber pull-out, and longitudinal cracking along the unreinforced core. Damage models indicate a brittle fracture behavior at the resin-rich surface, transitioning to ductile tearing and shear failure in the interior, and local buckling and void formation further compromised the load transfer mechanisms. Inadequate resin dispersion not only reduced the structural capacity of the composite but also introduced critical planes of weakness that triggered early failure under loading.

**Figure 13:**
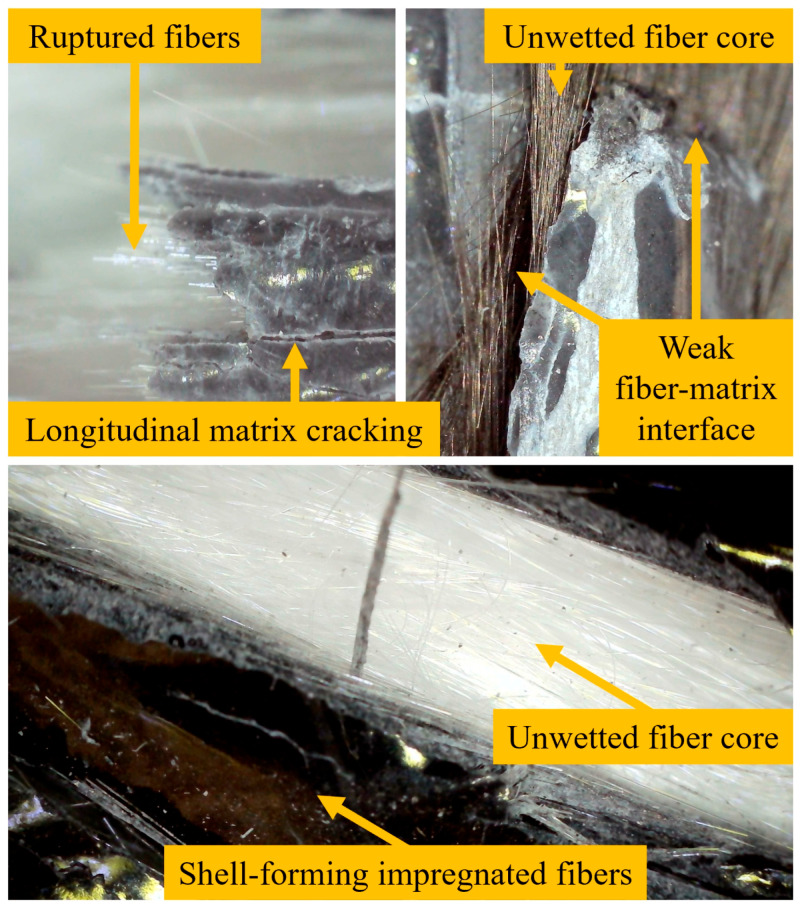
Microscope images of the fractured fiber rope composites.

In the samples reinforced with 2% GNPs, the post-failure microstructural examination reveals even more pronounced issues compared to the 1% GNP series. Although the outer surfaces again exhibit a relatively smooth and well-bonded resin-rich shell, the interior regions display severe resin starvation, particularly evident through the extensive presence of dry, unbonded carbon filaments. The elevated nanoparticle concentration may have increased the viscosity of the epoxy matrix, significantly reducing its ability to infiltrate deep into the fiber bundles. As a result, fiber wetting appears to be superficial and restricted primarily to the peripheral zones, leaving the central regions vulnerable to premature fiber pull-out and slippage under load. The fracture morphology is characterized by sharp interfacial delaminations, indicating poor stress transfer and weak fiber–matrix adhesion. Longitudinal cracking and fiber bundle separation are prevalent, suggesting a lack of cohesive integrity across the cross-section. The failure pattern confirms that excessive nanoparticle loading—despite improving local matrix properties—can hinder composite performance by compromising resin flow and distribution, ultimately creating weak zones that act as initiation sites for structural failure. Figure 13 shows a highly magnified view of the composite ropes after the mechanical experiments.

**Figure 14:**
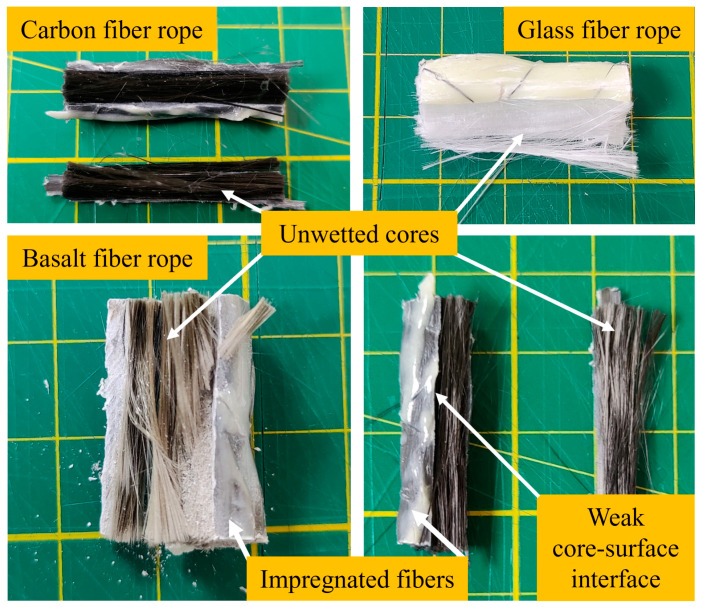
The cross-sectional images of fiber rope composites.

The cross-sectional images of the fabricated fiber rope composites, obtained by cutting fractured specimens, are shown in Figure 14. In these images, rope surfaces wetted with epoxy resin and unwetted core regions are seen. The disadvantage in the process of making composites from fiber ropes occurs in this part. As can be seen from the images, the epoxy resin is not able to penetrate into the core region. This causes only the rope surfaces to be cured with epoxy, resulting in a rigid and brittle structure, while the inner regions remain as flexible fibers. This flexibility is particularly apparent in the bending test, and the tendency toward deflection during the test is facilitated. As a result, fiber rope composites that show a flexible and ductile behavior in bending may be suitable for applications where these properties are desired.

## 4. Conclusions

Composite technology occupies a significant position in modern materials engineering, offering comprehensive solutions for applications that require high strength, lightweight structures, and effective structural reinforcement. These systems are particularly valuable in scenarios where enhanced durability and superior mechanical performance are critical. Recent advancements in nanotechnology have further extended the capabilities of composite materials through the integration of nanoscale reinforcements. Among them, graphene nanoplatelets (GNPs) have emerged as promising additives due to their high surface area and exceptional mechanical properties.

In this study, carbon, basalt, and glass fiber ropes were fabricated using an epoxy matrix modified with GNPs at 0.5%, 1%, and 2% weight fractions. The primary aim was to assess the influence of GNP modification on the mechanical behavior of composite ropes under various loading conditions, including flexural, compressive, and buckling scenarios. The results demonstrate that GNPs significantly enhance load transfer efficiency and delay the onset of microcracking when incorporated at optimal concentrations.

Experimental findings identified 0.5 wt% GNPs as the most effective reinforcement ratio, yielding consistent and homogeneous nanoparticle dispersion throughout the matrix, thereby improving the overall mechanical performance. In contrast, higher concentrations (1% and 2%) led to nanoparticle agglomeration and increased resin viscosity, which resulted in local stress concentrations and reduced structural integrity. These adverse effects were particularly evident under flexural and compressive loads.

In conclusion, this study confirmed that the judicious incorporation of GNPs at optimized levels can substantially improve the mechanical performance of fiber rope composites, with potential applications in structural strengthening and seismic retrofitting. In addition, it has been understood that the mechanical performance of fiber rope composites could be improved in field applications by using the matrix modification process with graphene nanostructures, which is a basic, simple, and easily applicable method. The findings provide a foundation for further exploration into nanostructure-enhanced composite systems, particularly in terms of optimizing particle distribution, immersion time, matrix compatibility, and fiber–matrix synergy. Future research should consider broader ranges of nanoparticle contents, alternative fiber types, and long-term durability evaluations to fully realize the potential of these materials in advanced engineering applications.

## Figures and Tables

**Table 1:** Density results of fiber rope composites (g/cm^3^).

	0% GNP	0.5% GNP	1% GNP	2% GNP
**GF**	1.85 ± 0.09	1.81 ± 0.03	1.81 ± 0.02	1.88 ± 0.02
**BF**	1.86 ± 0.07	1.87 ± 0.03	1.97 ± 0.02	1.53 ± 0.02
**CF**	1.54 ± 0.02	1.54 ± 0.01	1.59 ± 0.01	1.56 ± 0.01

**Table 2:** Mechanical test results of glass fiber rope composites.

	Flexural Strength (MPa)	Compressive Strength (MPa)	Buckling Peak Load (N)
**GF Rope Comp.**	133.88 ± 18.84	52.60 ± 4.49	1040.63 ± 161.04
**GF Rope Comp. −0.5% GNP**	147.97 ± 7.59	57.38 ± 2.64	1190.53 ± 162.85
**GF Rope Comp. −1% GNP**	130.77 ± 11.28	66.70 ± 6.60	1179.79 ± 124.13
**GF Rope Comp. −2% GNP**	117.80 ± 11.07	73.38 ± 2.79	808.59 ± 82.58

**Table 3:** Mechanical test results of basalt fiber rope composites.

	Flexural Strength (MPa)	Compressive Strength (MPa)	Buckling Peak Load (N)
**BF Rope Comp.**	125.66 ± 12.60	77.21 ± 5.05	1046.23 ± 45.27
**BF Rope Comp. −0.5% GNP**	123.81 ± 6.73	86.93 ± 7.27	1325.57 ± 60.33
**BF Rope Comp. −1% GNP**	112.02 ± 8.67	60.56 ± 10.98	1274.48 ± 99.76
**BF Rope Comp. −2% GNP**	114.54 ± 5.77	74.99 ± 7.48	1302.09 ± 186.98

**Table 4:** Mechanical test results of carbon fiber rope composites.

	Flexural Strength (MPa)	Compressive Strength (MPa)	Buckling Peak Load (N)
**CF Rope Comp.**	109.22 ± 4.32	46.50 ± 4.35	1369.54 ± 155.44
**CF Rope Comp. −0.5% GNP**	111.06 ± 7.16	53.08 ± 1.94	1470.32 ± 127.45
**CF Rope Comp. −1% GNP**	113.30 ± 7.09	58.45 ± 7.53	1226.57 ± 160.16
**CF Rope Comp. −2% GNP**	121.77 ± 7.05	61.73 ± 2.12	1115.63 ± 48.61

## Data Availability

The original contributions presented in this study are included in the article. Further inquiries can be directed to the corresponding author.

## References

[1] Aydın M.R., Acar V., Çakır F., Gündoğdu Ö. (2025). Effect of Manufacturing Parameters on Low-Velocity Impact Behavior of Aramid, Carbon and Glass Fiber Reinforced Polymer Composites Using Taguchi Experimental Design. Appl. Compos. Materials.

[2] Foster G.P. (2002). Advantages of Fiber Rope Over Wire Rope. J. Ind. Text..

[3] Monni F., Quagliarini E., Lenci S., Clementi F. (2015). Dry Masonry Strengthening Thotugh Basalt Fiber Ropes:Experimental Results Versus Out-Of-Plane Actions. Key Eng. Mater..

[4] Monni F., Quagliarini E., Lenci S. (2017). Pre-Tensioned Basalt Fibers Ropes Stitching for Masonry Strengthening against Vertical Bending: A First Experimental Insight. Key Eng. Mater..

[5] TRousakis C., Panagiotakis G.D., Archontaki E.E., Kostopoulos A.K. (2019). Prismatic RC columns externally confined with FRP sheets and pretensioned basalt fiber ropes under cyclic axial load. Compos. Part B.

[6] Phong N.H., Matsumoto A., Shimomura T., Sekijima K. (2006). Seismic Retrofitting of Reinforced Concrete Columns with Continuous Fiber Rope. Proc. Jpn. Concr. Inst..

[7] Pan J., Wang X., Dong H. (2021). Strengthening of Precast RC Frame to Mitigate Progressive Collapse by Externally Anchored Carbon Fiber Ropes. Polymers.

[8] Ali M. (2017). Seismic performance of coconut-fiber-reinforced-concrete columns with different reinforcement configurations of coconut-fiber ropes. Constr. Build. Mater..

[9] Ali M., Chouw N. (2013). Experimental investigations on coconut-fiber rope tensile strength and pullout from coconut fiber reinforced concrete. Constr. Build. Mater..

[10] Abdulla K.F., Cunningham L.S., Gillie M. (2021). Out-of-plane strengthening of adobe masonry using hemp fiber ropes: An experimental investigation. Eng. Struct..

[11] Hussain Q., Ruangrassamee A., Joyklad P., Wijeyewickrema A.C. (2022). Shear Enhancement of RC Beams Using Low-Cost Natural Fiber Rope Reinforced Polymer Composites. Buildings.

[12] Alraie A., Spadea S., Matsagar V. (2024). Enhanced sustainability through strengthening of existing structures with natural fiber ropes. Procedia Struct. Integr..

[13] Quagliarini E., Monni F., Lenci S., Bondioli F. (2012). Tensile characterization of basalt fiber rods and ropes: A first contribution. Constr. Build. Mater..

[14] Quagliarini E., Monni F., Bondioli F., Lenci S. (2016). Basalt fiber ropesandrods:Durabilitytestsfortheiruseinbuilding engineering. J. Build. Eng..

[15] Liang J., Wu C., Ping H., Wang M., Tang W. (2020). Surface Pretreatment and Fabrication Technology of Braided Carbon Fiber Rope Aluminum Matrix Composite. Metals.

[16] Sry V., Jung D.Y., Mizutani Y., Endo G., Todoroki A. (2021). Effect of preload treatment on elastic modulus of braided synthetic fiber rope for static loading. J. Text. Inst..

[17] Bain C., Davies P., Bles G., Marco Y., Barnet J. (2020). Influence of bedding-in on the tensile performance of HMPE fiber ropes. Ocean. Eng..

[18] Durmuş-Sayar A., Tansan M., Çinko-Çoban T., Serttan D., Dizman B., Yildiz M., Ünal S. (2024). Incorporation of Graphene Nanoplatelets into Fiber-Reinforced Polymer Composites in the Presence of Highly Branched Waterborne Polyurethanes. Polymers.

[19] Acar V., Erden S., Sarıkanat M., Seki Y., Akbulut H., Seydibeyoğlu M.Ö. (2020). Graphene oxide modified carbon fiber prepregs: A mechanical comparison of the effects of oxidation methods. Express Polym. Lett..

[20] Rafiee M.A., Rafiee J., Wang Z., Song H., Yu Z.Z., Koratkar N. (2009). Enhanced Mechanical Properties of Nanocomposites at Low Graphene Content. Acsnano.

[21] Zaman I., Phan T.T., Kuan H.C., Meng Q., La L.T.B., Luong L., Youssf O., Ma J. (2011). Epoxy/graphene platelets nanocomposites with two levels of interface strength. Polymer.

[22] Çakir F., Uysal H. (2015). Experimental modal analysis of brick masonry arches strengthened prepreg composites. J. Cult. Herit..

[23] Çakir F., Uysal H., Acar V. (2016). Experimental modal analysis of masonry arches strengthened with graphene nanoplatelets reinforced prepreg composites. Measurement.

[24] Acar V., Çakir F., Uysal H., Seydibeyoğlu M.Ö., Akbulut H., Mosalam K.M. (2017). Strengthening of concrete beams by monolayer prepreg composites with and without graphene reinforcement. Constr. Build. Mater..

[25] Cakir F., Acar V., Zulfikar A.C., Tutar A.I. (2025). A novel strengthening process for masonry tower-type structures with irregular geometry using carbon fiber composite ropes. Bull. Earthq. Eng..

[26] Technical Datasheet Teknobond 330 Epoxy Based Adhesive and Lamination Resin, Tekno Construction Chemicals. https://www.teknoyapi.com.tr/en/products/construction-reinforcement-products/teknobond-330-epoxy-based-adhesive-and-lamination-resin.

[27] Chen L., Zhao Y., Li M., Li L., Hou L., Hou H. (2021). Reinforced AZ91D magnesium alloy with thixomolding process facilitated dispersion of graphene nanoplatelets and enhanced interfacial interactions. Mater. Sci. Eng. A.

[28] (2013). Standard Test Methods for Density and Specific Gravity (Relative Density) of Plastics by Displacement.

